# Understanding effects of engaging online learning environments on students’ cognitive engagement and well-being: the role of academic self-concept and flow

**DOI:** 10.3389/fpsyg.2025.1614109

**Published:** 2025-10-10

**Authors:** Yafei Shi, Mengjin Chen, Yantao Wei, Junli Shen, Mingyue Wu, Ke Zhu

**Affiliations:** ^1^Faculty of Education, Henan Normal University, Xinxiang, Henan, China; ^2^Henan Collaborative Innovation Center for Intelligent Education, Henan Normal University, Xinxiang, Henan, China; ^3^Faculty of Artificial Intelligence in Education, Central China Normal University, Wuhan, Hubei, China

**Keywords:** engaging online learning environments, cognitive engagement, well-being, academic self-concept, flow

## Abstract

Engaging online learning environments (EOLE) is one of the most critical drivers of students’ engagement in online learning. However, discussions about how and to what extent EOLE affects students’ engagement are under-researched. Therefore, it is necessary to explore the effect of EOLE on students’ cognitive engagement. In this study, 368 graduate students enrolled in online courses were surveyed. The partial least square structural equation modeling was employed to explore the relationships among EOLE, academic self-concept, flow, cognitive engagement and well-being. Results showed that EOLE had positive effects on academic self-concept, flow, deep and shallow cognitive engagement, and well-being. In addition, academic self-concept mediated the effect of EOLE on well-being, but its mediating role between EOLE and both deep and shallow cognitive engagement was not observed. Flow mediated the effect of EOLE on both deep and shallow cognitive engagement, but its mediating role between EOLE and well-being was not significant. Furthermore, the effects of EOLE on both deep and shallow cognitive engagement were sequentially mediated through academic self-concept and flow. However, the sequential mediating effects of academic self-concept and flow in the relationship between EOLE and well-being were also not significant. This study suggests that practitioners and educators should design effective online learning environments to improve online learners’ engagement and well-being.

## 1 Introduction

Recent years have seen increased availability and adoption of online learning across all levels of education (Lockee, 2021). Online learning offers more benefits to students, including flexibility of time and place, accessibility and efficiency (Jiang et al., 2024; Salas-Pilco et al., 2022), and opportunities for students who are unable to attend face-to-face classes due to work, family commitments or other urgent matters (Heo et al., 2021; Shi et al., 2024). Since the COVID-19 pandemic, educators have increasingly recognized the importance of online learning for students (Alon et al., 2023). It provides learners with a continuous, learner-centered educational environment, and thus has been extensively adopted by higher education institutions worldwide (Wang et al., 2022). Moreover, recent evidence shows that online learning could achieve comparable and better learning achievements compared to traditional in-person learning in higher education (Alarifi and Song, 2024). Therefore, designing engaging online learning environments by educators and practitioners is crucial for students’ academic performance.

However, online learning also faces some challenges, such as insufficient teacher support, lack of motivation and engagement, issues of psychological well-being, and technical obstacles (Barrot et al., 2021; Chiu et al., 2021; Fiddiyasari and Pustika, 2021; Zhou and Yu, 2021). Researchers claimed that levels of students’ engagement, motivation, and course completion in online learning environments are lower than those in traditional educational settings (Stark, 2019; Wang et al., 2023). Furthermore, diminishing motivation and elevated workload in online learning settings may subsequently lead to compromised well-being (Slack and Priestley, 2023). Learning engagement is a multidimensional concept, encompassing behavioral, emotional, and cognitive engagement (Fredricks et al., 2004). Cognitive engagement is an important component of student engagement, which involves high-level knowledge construction and meaningful processing strategy (Guo et al., 2023; Salas-Pilco et al., 2022; Shi et al., 2021). Well-being involves all the ways people positively experience and evaluate their lives (Tov, 2018). A few studies have explored the relationship between online learning environments and cognitive engagement, and well-being. For instance, Liu and Duan’ s (2022) research findings have indicated that students’ self-, peer-, and technological factors in online learning environments can significantly enhance individual deep and shallow cognitive engagement. An empirical study shows that, in online learning environments, perceived social support is positively correlated with life satisfaction and positive emotions among Chinese college students, while negatively correlated with negative emotions (Huang and Zhang, 2022). Although previous studies have shown that the quality of online learning environments positively affects students’ cognitive engagement and well-being, there is a scarcity of studies examining the impacts of online learning environments on students’ cognitive engagement and well-being with empirical research method in higher education contexts.

Academic self-concept is the students’ self-evaluation of their competence in a particular field (Schnitzler et al., 2021). Academic self-concept is considered to be associated with various desirable educational outcomes, such as academic performance and satisfaction (Wu et al., 2021; Zhan and Mei, 2013). Students with higher academic self-concept tend to choose more challenging learning environments, because it can stimulate students’ interest and enthusiasm for learning, thereby enhancing their satisfaction (Zhan and Mei, 2013). However, students may show lower self-concept in online learning environments due to the limited interaction and support provided by online contexts (Bringula et al., 2021; Zhan and Mei, 2013). Existing research on academic self-concept primarily focuses on traditional learning environments and children or adolescents, few studies exploring the impact of online learning environments on academic self-concept in higher education contexts (Guo et al., 2022; Steinberg et al., 2024). Evidence shows that academic self-concept is closely related to cognitive learning strategies (Lohbeck and Moschner, 2022) and well-being (Céspedes et al., 2021). For instance, Zhang et al. (2022) have demonstrated that academic self-concept positively influences deep learning in online learning environments. Similarly, the research findings of Coutts et al. (2023) have revealed that self-concept is associated with well-being indicators (lower depressive symptoms and perceived stress, and higher satisfaction with life). Flow refers to the psychological experience of immersion in an activity and a sense of control over the surrounding environment (Esteban-Millat et al., 2014). In online learning contexts, students’ perceptions of skills and challenges are important factors in determining their level of flow (Shin, 2006). Spefically, when the challenge level is equal to or slightly higher than perceived skill, a flow experience may occur (Shin, 2006). This indicates a potential relationship between academic self-concept and flow (Lesmana, 2019). However, research exploring the relationship between academic self-concept and flow remains in its infancy. In addition, flow is another essential factor influencing cognitive engagement and well-being (Landhäußer and Keller, 2012; Mao et al., 2024). A high level of concentration and enjoyment in the learning activity enables students to create a state of flow (Czikszentmihalyi, 1990; Zhang et al., 2024). Conversely, students may exhibit a low level of cognitive engagement when they experience a lack of interest and enjoyment from the learning activity (Schnitzler et al., 2021). Therefore, flow could promote positive affect and well-being (Mao et al., 2024). In addition, academic self-concept affects students’ motivation (Wu and Kang, 2023), which in turn influences flow (Suryaratri et al., 2022). For instance, the higher the students’ perceived competence, the more likely they are to experience enjoyment and show cognitive engagement (Schnitzler et al., 2021). Although previous studies have shown that academic self-concept and flow are crucial for students’ engagement and well-being, the role of academic self-concept and flow in the effect of online learning environments on cognitive engagement and well-being remains underexplored.

To address the mentioned gaps, this study examines the effects of online learning environments on cognitive engagement and well-being and explores the sequential mediating effects of academic self-concept and flow in this relationship. This study sheds light on the design and implementation of an engaged and friendly online learning environment for practitioners and researchers.

## 2 Theoretical framework

Social cognitive theory (SCT) emphasizes the understanding of human behavior through the interactive influence of the environment, person, and behavior (Bandura, 1991). SCT believes that people learn by observing others and the effects of their behavior, as well as through direct interaction with people or technology. Therefore, it is also called triadic reciprocal determinism (Shahzad et al., 2025). SCT has been widely applied within psychological disciplines as well as in other fields such as education, business, and health (Schunk and DiBenedetto, 2020). In educational settings, environmental factors are seen as elements that are physically external to the individual and that provide opportunities and social support for students to learn (Carillo, 2010). Personal factors involve cognitions, beliefs, skills, and affect (Schunk and Usher, 2012). Behavioral factors are related to components such as task selection, effort, persistence, and effective learning strategies (Schunk and DiBenedetto, 2016). These three factors may be positively related to perceived and actual learning effectiveness at different stages of the learning process (Lin et al., 2024). Personal factors engage in a dynamic and reciprocal interaction with both behaviors and environments (Schunk and DiBenedetto, 2020). Based on SCT, this study conceptualizes these three distinct dimensions: environmental factors as online learning environments, individual factors as academic self-concept and flow state, and behavioral factors as students’ cognitive engagement and well-being.

### 2.1 Engaging online learning environments

Although online learning is widely applied in higher education due to its significant advantages (Wang et al., 2022), it has long faced numerous challenges, with some of the major issues being high dropout rates, lack of motivation, and low levels of engagement (Hoi, 2022; Stark, 2019). Many reported challenges are related to interpersonal relationships and course quality in online learning environments, such as teaching presence, teacher support, and instructional strategies (Heilporn et al., 2022; Pan, 2022; Turk et al., 2022). Therefore, researchers have conducted extensive investigations focusing on online learning environments where learning engagement is facilitated or inhibited by certain factors (Hoi, 2022). For instance, Gray et al. (2016) found that peer interaction and teacher presence could significantly contribute to student engagement in online learning environments. Cole et al. (2021) examined the predicting role of student active learning practices and online learning climate (e.g., course structure and student connectedness) on engagement in online learning environments. In addition, Heilporn et al. (2022) suggested that instructional strategies (e.g., trusting relationships, the relevance of activities, content, resources, and course pace) were significant antecedents of student engagement in online learning. Therefore, this study employed the framework of engaging online learning environments (EOLE) proposed by Hoi (2022) featuring course clarity, student connectedness, course structure, provision of choice, teaching relevance, teacher emotional support, and teacher presence.

### 2.2 Cognitive engagement and well-being in EOLE

Cognitive engagement is defined as the extent to which individuals apply sophisticated learning strategies when undertaking a learning task (Chiu, 2022). According to the levels of processing theory (Craik and Lockhart, 1972), cognitive engagement consists of two distinct categories: deep and shallow (Greene and Miller, 1996). Deep learning emphasizes individual understanding, constructing, reflecting, transferring, and implementing new knowledge (Zhang et al., 2022). In contrast, shallow processing involves only superficial analysis of information (e.g., rote learning) (Sugden et al., 2021). Prior research has shown that although deep and shallow cognitive engagement have opposite effects on learning performance, both deep and shallow cognitive engagement can facilitate learning in various contexts (Greene and Miller, 1996). A higher level of cognitive engagement leads to better academic performance in online learning environments (Guo et al., 2023). Some researchers believe that students who are engaged in a task or discussion will employ more advanced cognitive processing strategies (Buijs and Admiraal, 2013; Liu et al., 2023). When the learning environment (e.g., teacher support, student cohesion, equity, and interaction) supports student learning, students are more likely to exhibit cognitive engagement (Guo et al., 2023; Tas, 2016). For instance, based on the PST model, Lin et al. (2022) explored the factors influencing students’ cognitive engagement in online learning environments. The results indicated that educational affordances positively enhance both deep and shallow cognitive engagement, while social and technological affordances significantly increase only shallow cognitive engagement. Nevertheless, how EOLE influences students’ cognitive engagement remains poorly explored.

Well-being is another important concept that is closely related to students’ learning and lives. The field of positive psychology has long established the three dimensions of well-being, namely, subjective well-being, social well-being, and psychological well-being (Murphy, 2023). Subjective well-being is assessed as a triadic structure that includes a person’s high positive emotions, low negative emotions, and high satisfaction with life (Deci and Ryan, 2008; Diener, 1984; Huang and Zhang, 2022). Social well-being is described as five dimensions, named integration, contribution, coherence, actualization, and acceptance (Keyes, 1998). Long-term psychological well-being does not mean that individuals feel good all the time, but rather that they can manage long-term negative emotions (Huppert, 2009). Evidence shows that the learning environment is one of the most important factors influencing student’s well-being (Wasson et al., 2016). For instance, Zhu and Van Winkel (2016) found that EOLE offered students with sickness the opportunity to continue their studies and interact with the school, which contributed to their mental well-being. In addition, online learning increases the time students spend on technological devices and sleep, while decreasing time spent on physical activities, potentially leading to reduced students’ well-being (Cockerham et al., 2021). Therefore, it is essential to explore what influences cognitive engagement and well-being in online learning environments for developing appropriate pedagogical strategies to enhance students’ online learning experience and performance. Therefore, the Hypothesis was proposed as follows:

*H1*: EOLE has a positive impact on (a) deep cognitive engagement, (b) shallow cognitive engagement, and (c) well-being.

### 2.3 The mediating role of academic self-concept on the link between EOLE and, cognitive engagement and well-being

Self-concept could be classically divided into two components: academic and non-academic (Marsh and Shavelson, 1985). Academic self-concept refers to the mental representation of an individual’s ability in academic and school subjects (Brunner et al., 2010). Recently, the academic self-concept has become an important educational research topic because it significantly relates to many desired academic outcomes (Arens et al., 2021). High-quality learning environments may contribute to students’ self-perception (Rohan et al., 2022). When individuals reflect on their experiences in authentic learning environments, academic self-concept is formed (Chen et al., 2021). Therefore, it is necessary to understand students’ academic self-concept, as it forms the foundation for fostering their interest in learning (Bringula et al., 2021). However, few studies have explored the effect of EOLE on academic self-concept (Rohan et al., 2022).

Academic self-concept is also associated with many desirable outcomes, such as academic performance, motivation, engagement, and well-being (Guo et al., 2022; Arens et al., 2021; Wu and Kang, 2023; Céspedes et al., 2021). For instance, Schnitzler et al. (2021) found that the higher the students’ perceived competence, the more likely they would be cognitively engaged. Buhs (2005) suggested that social support from learning environments influences students’ perceptions of competence, which in turn promotes their engagement. In addition, academic self-concept is a major predictor of well-being (Céspedes et al., 2021). Previous studies have shown that the self-concept of adolescents was positively correlated with the positive dimensions of their well-being (e.g., positive affect and life satisfaction), but not with negative affect (McCullough et al., 2000). Therefore, this study hypotheses that.

*H2*: EOLE indirectly impacts (a) deep and (b) shallow cognitive engagement, and (c) well-being through academic self-concept.

### 2.4 The mediating role of flow on the link between EOLE and, cognitive engagement and well-being

Flow refers to a highly focused state of complete absorption in an activity, which is characterized by intense focus, control, interest, and a balance between skills and challenges (Csikszentmihalyi and Csikzentmihaly, 1990; Goh and Yang, 2021). When a student is cognitively engaged in a learning activity, it would result in a positive flow experience (Czikszentmihalyi, 1990; Landhäußer and Keller, 2012; Mao et al., 2024; Suryaratri et al., 2022). Therefore, a lack of autonomy experience reduces the likelihood of an individual entering a flow state (Barthelmäs and Keller, 2021). Studies have shown that some activities can induce a flow tendency to a certain extent (Pritikin and Schmidt, 2022). In educational contexts, flow could foster motivation, cognitive ability, academic performance, and creativity (Csikszentmihalhi, 2020).

Studies found that EOLE was significantly associated with flow state (Esteban-Millat et al., 2014). For instance, Özhan and Kocadere (2020) showed that flow is one of the important factors for academic success in gamified online learning environments. On the other hand, cognitive engagement is influenced by the flow state in online learning (Landhäußer and Keller, 2012). For instance, De Manzano et al. (2010) indicate that a flow state could be experienced when deeply engaged and highly focused on a task. Furthermore, a positive flow experience is important for happiness and well-being (Bonaiuto et al., 2016). However, some studies have also found no significant link between flow and well-being. For instance, Rheinberg et al. (2007) observe that the flow experience at work is not highly correlated with well-being. Therefore, the relationship between flow and well-being needs to be further explored in online educational contexts. Therefore, the hypothesis was proposed:

*H3*: EOLE indirectly impacts (a) deep and (b) shallow cognitive engagement, and (c) well-being through flow.

### 2.5 The sequential mediating effects of academic self-concept and flow on the relationship between EOLE and, cognitive engagement and well-being

Perceived ability is a general assessment of self-concept in a specific situation (Jackson et al., 2001). One of the conditions for creating flow is a balance between task difficulty and perceived abilities (De Manzano et al., 2010). If students are not confident in his or her ability, it is difficult to get into the flow state (Lesmana, 2019). This means that passive activities do not involve a skill component and therefore are not associated with the flow experience (Barthelmäs and Keller, 2021). Consequently, the academic self-concept is a prerequisite for flow. Relevant research has shown that flow could be a driver of student engagement in the classroom (Lesmana, 2019), and self-concept and self-efficacy are factors in the development of flow (Jackson et al., 2001; Mesurado et al., 2016). Therefore, we assume that:

*H4*: Academic self-concept and flow sequentially mediate the relationship between EOLE and (a) deep and (b) shallow cognitive engagement, and (c) well-being.

**Figure 1:**
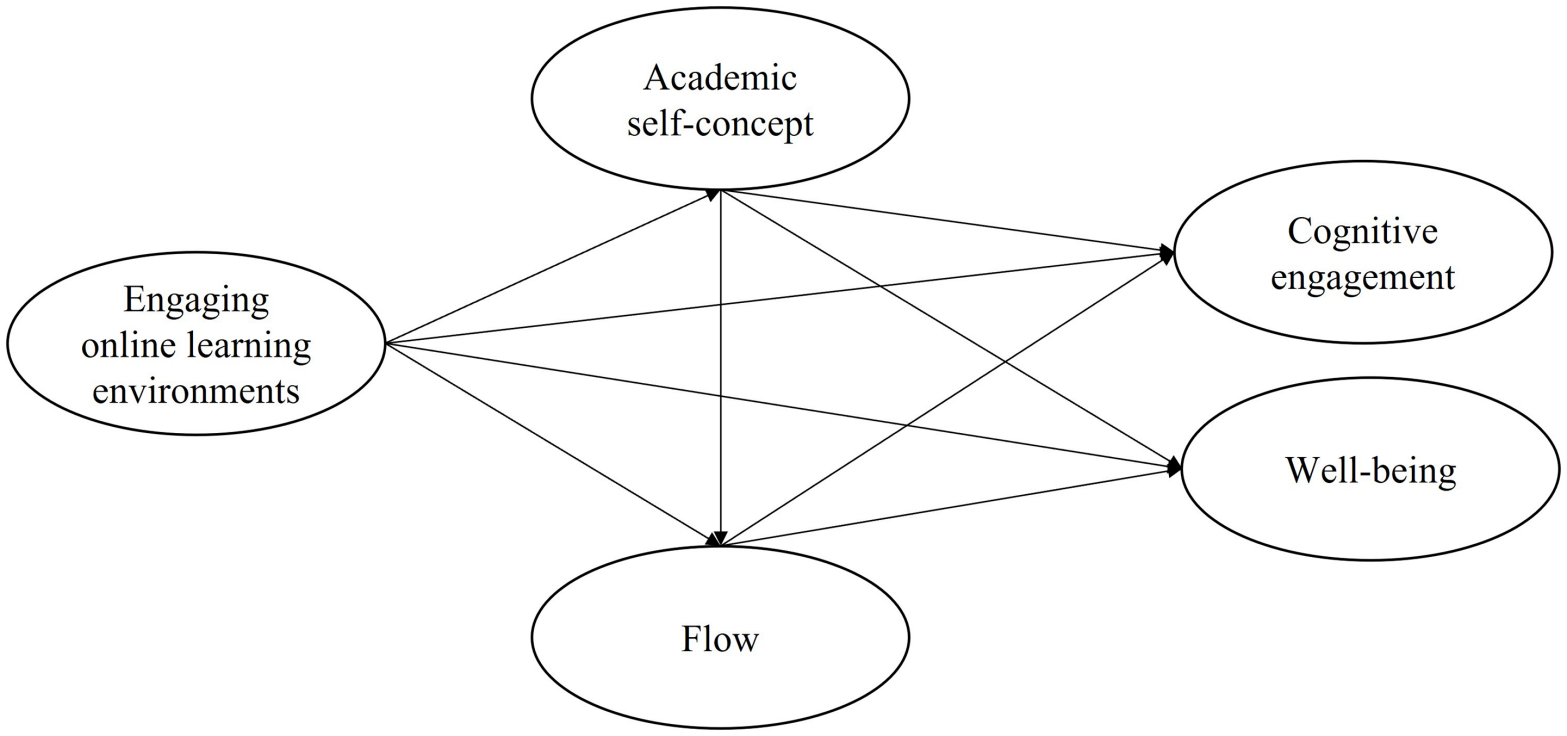
The proposed research model.

In sum, the hypothetical model is presented in Figure 1. It was proposed that EOLE not only influenced cognitive engagement and well-being directly but also through the mediating role of academic self-concept and flow.

## 3 Methods

### 3.1 Participants and procedure

Participants in this study consisted of graduate students who enrolled in the two-month online course “Research Ethics and Academic Standards,” offered by Henan Normal University. Students who completed this course voluntarily elected to participate in the study. The questionnaire was distributed through WJX tools^1^ in Winter 2024. Before completing the questionnaire, all participants were informed of the purpose of the survey and participated voluntarily and anonymously. This study has been approved by our university’s Research Ethics Committee. Then, participants were required to complete a series of questionnaires, including demographic information and the items assessing EOLE, academic self-concept, flow, deep and shallow cognitive engagement, well-being. Finally, a total of 368 graduate students were surveyed. After receiving the data, we eliminated 31 students who provided invalid responses and the valid response rate was 91.58%. As shown in Table 1, the average age of remaining participants was about 23 with a range from 19 to 28 years. Males and females account for 46.9% (*n* = 158) and 53.1% (*n* = 179) respectively. 52.5% (*n* = 177) of them are in the first year of graduate studies, 40.9% (*n* = 138) in the second year, and 6.5% (*n* = 22) in the third year.

**Table 1:** Respondents profile.

Demographics	Number	Percent (%)
Gender		
Male	158	46.9
Female	179	53.1
Age		
19	2	0.6
21	10	3.0
22	84	24.9
23	110	32.6
24	74	22.0
25	36	10.7
26	8	2.4
27	4	1.2
28	9	2.7
Grade		
1	177	52.5
2	138	40.9
3	22	6.5
Ranking		
Upper middle	139	41.2
Middle	144	42.7
Lower middle	54	16.0
Region		
Urban	111	32.9
Rural	226	67.1

### 3.2 Instruments

The questionnaire was designed in two parts. The first part was to collect participants’ demographic information. The other was employed to collect participant’s perceptions including EOLE, academic self-concept, flow, deep and shallow cognitive engagement, and well-being. First, the questionnaire was translated into Chinese by two doctoral students. Second, a researcher with 9 years of rich teaching and research experience refined the language to make it more precise.

#### 3.2.1 EOLE scale

22 items of the EOLE scale were adapted from Hoi (2022) which consists of seven dimensions including course clarity (4 items), student connectedness (3 items), course structure (3 items), provision of choice (3 items), teacher relevance (3 items), teacher emotional support (3 items), and teacher presence (3 items). Sample items were, “The organization of the course was clear” and “student as comfortable with one another.” The questionnaire is a five-point Likert scale ranging from 1 (strongly disagree) to 5 (strongly agree). The internal consistency coefficients (Cronbach’s alpha, *α*) were 0.954 for course clarity, 0.947 for student connectedness, 0.942 for course structure, 0.933 for provision of choice, 0.935 for teacher relevance, 0.940 for teacher emotional support, and 0.932 for teacher presence. The overall α coefficient of this EOLE scale was 0.980.

#### 3.2.2 Academic self-concept scale

All 8 items of the academic self-concept scale were adapted from Matovu (2014) which involves academic confidence (3 items) and academic effort (5 items). The sample items were “If I work hard, I think I can get better grades” and “I often do my course work without thinking.” This questionnaire is a seven-point Likert scale ranging from 1 (strongly disagree) to 7 (strongly agree). The internal consistency α coefficients were 0.952 for academic confidence and 0.965 for academic effort, respectively. The overall α coefficient of this scale was 0.962.

#### 3.2.3 Flow scale

All 6 items of the flow scale were adapted from the flow experience (Park et al., 2010). One of the items was that “Taking an online course excited my curiosity.” Example items include, “The online course allowed me to control the whole learning process” and “Interacting with the online course made me curious.” The questionnaire is a five-point Likert scale ranging from 1 (strongly disagree) to 5 (strongly agree). The internal consistency α coefficient of this scale was 0.965.

#### 3.2.4 Cognitive engagement scale

All 7 items of the cognitive engagement scale were adapted from Miller et al. (1996) which consists of deep strategy use (4 items) and shallow processing strategy use (3 items). Example items were “I work practice problems to check my understanding of new concepts or rules” and “When I study for tests I review my class notes and look at solved problems.” This is a five-point Likert ranging from 1 (strongly disagree) to 5 (strongly agree). The α coefficient of this scale was 0.960 for deep strategy use and 0.942 for shallow processing strategy use, respectively, and the overall α value was 0.974.

#### 3.2.5 Well-being scale

Three items of the well-being scale were adapted from Diener et al. (2010). The questionnaire is a seven-point Likert ranging from 1 (strongly disagree) to 7 (strongly agree). Sample items were “I lead a purposeful and meaningful life” and “My social relationships are supportive and rewarding.” The internal consistency α coefficient of this scale was 0.955.

### 3.3 Data analysis

The collected data were analyzed by SPSS 25 and Smart PLS 3 tools. First, the consistency coefficients of scales were calculated using the SPSS tool. Second, confirmatory factor analysis was tested to ensure good indicator reliability. Finally, the structural equation modeling was conducted through Smart PLS to evaluate the hypothesized model. Partial Least Squares Structural Equation Modelling (PLS-SEM) was employed in this study. Compared to covariance-based SEM techniques, PLS-SEM is more flexible, which works particularly well with small sample sizes (Hair et al., 2012). More importantly, PLS-SEM is well suited for exploratory research and theory development, as in the present study exploring sequentially mediate effects of academic self-concept and flow in the effects of EOLE on students’ cognitive engagement and well-being (Shi et al., 2024).

## 4 Results

### 4.1 Preliminary analysis

Skewness and kurtosis were employed to evaluate the normal distribution of the data. According to Lu et al. (2023), absolute values of skewness and kurtosis greater than 3.0 and 8.0, respectively, were defined as cutoff values for nonnormality. In this study, all measurements were within these ranges (skewness values between −1.682 and 0.928, and Kurtosis values between −1.996 and 2.630), indicating that all measured variables demonstrate normal distribution. As shown in Table 2, Pearson’s correlation analyses indicated that engaging online learning environments, academic self-concept, flow, cognitive engagement, and well-being were significantly correlated.

**Table 2:** Descriptive statistic and Pearson correlation.

	CC	SC	CS	POC	TR	TES	TP	AC	AE	Flow	CED	CES	WB
CC	1												
SC	0.926^**^	1											
CS	0.862^**^	0.893^**^	1										
POC	0.837^**^	0.847^**^	0.909^**^	1									
TR	0.872^**^	0.878^**^	0.894^**^	0.874^**^	1								
TES	0.853^**^	0.834^**^	0.862^**^	0.848^**^	0.925^**^	1							
TP	0.835^**^	0.839^**^	0.876^**^	0.840^**^	0.912^**^	0.931^**^	1						
AC	0.811^**^	0.801^**^	0.853^**^	0.814^**^	0.881^**^	0.863^**^	0.857^**^	1					
AE	0.814^**^	0.797^**^	0.827^**^	0.820^**^	0.866^**^	0.848^**^	0.835^**^	0.926^**^	1				
Flow	0.815^**^	0.793^**^	0.797^**^	0.791^**^	0.848^**^	0.812^**^	0.810^**^	0.843^**^	0.877^**^	1			
DCE	0.813^**^	0.796^**^	0.814^**^	0.797^**^	0.840^**^	0.818^**^	0.823^**^	0.829^**^	0.810^**^	0.840^**^	1		
SCE	0.798^**^	0.764^**^	0.783^**^	0.763^**^	0.827^**^	0.801^**^	0.796^**^	0.794^**^	0.809^**^	0.831^**^	0.938^**^	1	
WB	0.790^**^	0.789^**^	0.779^**^	0.762^**^	0.815^**^	0.770^**^	0.782^**^	0.833^**^	0.830^**^	0.792^**^	0.870^**^	0.879^**^	1
Min	3	3	3	3	3	3	3	4	4	3	3	3	4
Max	5	5	5	5	5	5	5	7	7	5	5	5	7
Mean	4.612	4.611	4.631	4.591	4.615	4.622	4.634	6.532	6.488	4.526	4.582	4.572	6.472
SD	0.533	0.539	0.521	0.545	0.519	0.530	0.519	0.701	0.711	0.566	0.551	0.551	0.747
VAR	0.284	0.291	0.271	0.297	0.270	0.281	0.269	0.492	0.505	0.320	0.304	0.304	0.558
Skewness	−1.127	−1.071	−1.136	−1.050	−1.032	−1.170	−1.151	−1.682	−1.590	−0.870	−1.067	−0.997	−1.513
Kurtosis	0.378	0.121	0.387	0.169	0.104	0.531	0.412	2.630	2.497	−0.186	0.265	0.083	1.860

**p < 0.01. CC, course clarity; SC, student connectedness; CS, course structure; POC, provision of choice; TR, teaching relevance; TES, teacher emotional support; TP, teacher presence; AC, academic confidence; AE, academic effort; DCE, deep cognitive engagement; SCE, shallow cognitive engagement; WB, well-being.

### 4.2 Assessment of measurement model

To assess the composite reliability and convergence validity, indicator loadings, composite reliability (CR), and average variance extracted (AVE) were employed. The two-stage approach was used to evaluate higher-order constructs in measurement model assessment (Sarstedt et al., 2019). In the first stage, lower-order constructs were evaluated. The results of composite reliability and convergence validity are shown in Table 3. All indicator loadings were greater than 0.8, and all *p*-values were statistically significant (*p* < 0.001), indicating that the indicator reliability was established. The composite reliability and convergence validity of the lower-order constructs were established in this study, with CR (0.956–0.973) and AVE (>0.8). The discriminant validity is presented in Table 4. The correlation coefficients between each latent construct were less than the square root of AVE, indicating the lower-order constructs had discriminant validity.

**Table 3:** The reliability and convergence validity of the measurement model in the first stage.

Constructs		Significance Test of Parameters				Composite Reliability	Convergence Validity
		Estimate	STDEV	*t*	*p*	CR	AVE
CC	CC1	0.942	0.012	76.274	***	0.967	0.880
	CC2	0.939	0.012	79.187	***		
	CC3	0.935	0.012	78.082	***		
	CC4	0.935	0.012	77.002	***		
SC	SC1	0.951	0.009	102.051	***	0.966	0.904
	SC2	0.955	0.008	117.158	***		
	SC3	0.947	0.010	97.943	***		
CS	CS1	0.951	0.010	91.027	***	0.963	0.896
	CS2	0.928	0.013	68.777	***		
	CS3	0.961	0.009	104.97	***		
POC	POC1	0.950	0.009	100.463	***	0.957	0.882
	POC2	0.927	0.012	74.366	***		
	POC3	0.940	0.011	81.822	***		
TR	TR1	0.950	0.009	101.833	***	0.959	0.885
	TR2	0.924	0.015	60.348	***		
	TR3	0.948	0.010	98.727	***		
TES	TES1	0.952	0.009	101.231	***	0.961	0.892
	TES2	0.935	0.012	80.267	***		
	TES3	0.946	0.014	68.909	***		
TP	TP1	0.935	0.017	54.12	***	0.956	0.880
	TP2	0.927	0.014	68.379	***		
	TP3	0.952	0.010	96.277	***		
AC	AC1	0.961	0.009	109.978	***	0.969	0.912
	AC2	0.946	0.013	70.995	***		
	AC3	0.959	0.010	93.909	***		
AE	AE1	0.889	0.031	28.605	***	0.973	0.877
	AE2	0.934	0.014	64.753	***		
	AE3	0.949	0.011	86.839	***		
	AE4	0.953	0.010	96.297	***		
	AE5	0.957	0.009	110.754	***		

***p < 0.001; CC, course clarity; SC, student connectedness; CS, course structure; POC, provision of choice; TR, teaching relevance; TES, teacher emotional support; TP, teacher presence; AC, academic confidence; AE, academic effort; STDEV, standard deviation; AVE, average variance extracted.

**Table 4:** The discriminant validity of the measurement model in the first stage.

Constructs	AC	AE	CC	CS	POC	SC	TES	TP	TR
AC	**0.955**								
AE	0.927	**0.937**							
CC	0.811	0.818	**0.938**						
CS	0.854	0.828	0.862	**0.947**					
POC	0.815	0.819	0.838	0.910	**0.939**				
SC	0.800	0.800	0.926	0.893	0.847	**0.951**			
TES	0.864	0.850	0.853	0.862	0.849	0.834	**0.945**		
TP	0.857	0.837	0.835	0.876	0.840	0.839	0.931	**0.938**	
TR	0.881	0.868	0.872	0.895	0.874	0.878	0.926	0.912	**0.941**

Values of diagonal represent the square root of AVE.

In the second stage, the higher-order constructs were evaluated (see Table 5). All indicator loadings, CR, and AVE values exceed the suggested threshold. Therefore, the composite reliability and convergence validity of the constructs in the second stage were adequate. The discriminant validity in the second stage is shown in Table 6. The correlation coefficients between each latent construct indicated a well-discriminant validity in the second stage.

**Table 5:** The reliability and convergence validity of the measurement model in the second stage.

Constructs		Significance test of parameters				Composite reliability	Convergence validity
		Estimate	STDEV	*t*	*p*	CR	AVE
EOLE	CC	0.935	0.013	73.035	***	0.983	0.892
	SC	0.940	0.012	77.17	***		
	CS	0.952	0.009	105.878	***		
	POC	0.931	0.018	51.894	***		
	TR	0.962	0.007	146.205	***		
	TES	0.947	0.010	91.655	***		
	TP	0.944	0.010	90.951	***		
ASC	AC	0.981	0.003	283.163	***	0.981	0.963
	AE	0.982	0.003	283.637	***		
Flow	Flow1	0.881	0.018	49.784	***	0.972	0.851
	Flow2	0.922	0.014	68.247	***		
	Flow3	0.929	0.014	67.649	***		
	Flow4	0.937	0.011	82.683	***		
	Flow5	0.947	0.009	109.883	***		
	Flow6	0.917	0.016	56.368	***		
DCE	DCE1	0.939	0.011	85.145	***	0.971	0.892
	DCE2	0.928	0.013	69.315	***		
	DCE3	0.952	0.010	97.173	***		
	DCE4	0.958	0.008	119.368	***		
SCE	SCE1	0.936	0.012	76.607	***	0.963	0.897
	SCE2	0.944	0.010	93.257	***		
	SCE3	0.962	0.007	139.632	***		
WB	WB1	0.953	0.008	113.785	***	0.971	0.918
	WB2	0.955	0.008	118.06	***		
	WB3	0.966	0.007	147.38	***		

***p < 0.001. EOLE, engaging online learning environment; ASC, academic self-concept; DCE, deep cognitive engagement; SCE, shallow cognitive engagement; WB, well-being; STDEV, standard deviation; AVE, average variance extracted.

**Table 6:** The discriminant validity of the measurement model in the second stage.

Constructs	ASC	CED	CES	ENO	Flow	WB
ASC	**0.982**					
DCE	0.836	**0.944**				
SCE	0.819	0.939	**0.947**			
EOLE	0.902	0.863	0.838	**0.944**		
Flow	0.877	0.842	0.832	0.859	**0.922**	
WB	0.848	0.870	0.880	0.830	0.792	**0.958**

Values of diagonal represent the square root of AVE.

### 4.3 Assessment of structural model

**Figure 2:**
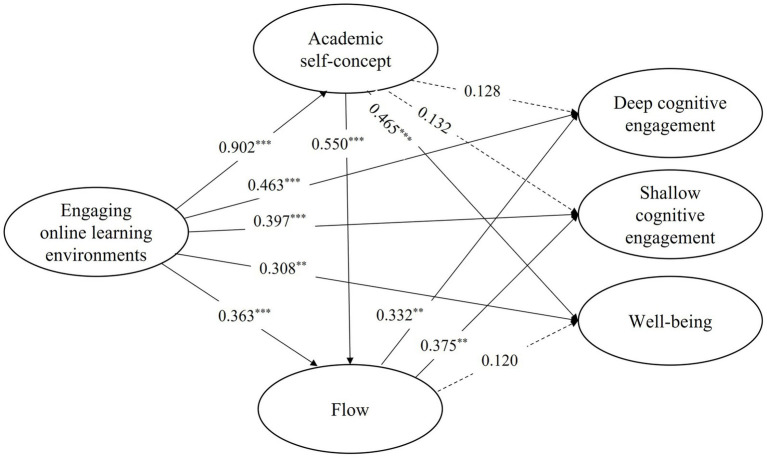
Results of hypothesis testing.

The fit of the structural model was evaluated using *R*^2^, *f*^2^, and *Q*^2^. The recommended cut-off thresholds are *R*^2^ > 0.19 (Chin, 1998), *f*^2^ > 0.02 (Chin, 1998), and *Q*^2^ > 0 (Sarstedt et al., 2021). As shown in Tables 7, 8, all values of *Q*^2^ are greater than 0.6, which suggests that the predictive relevance of the structural model is strong. All endogenous variables of R^2^ range from 0.745 to 0.814, which means that endogenous latent variables are adequately explained. The significant level of standardized path coefficients was examined through the bootstrapping method with 5,000-time sampling at the confidence level of 95%. As presented in Table 7 and Figure 2, EOLE significantly predicted academic self-concept (*β* = 0.902, *t* = 60.45, *p* < 0.001), deep cognitive engagement (*β* = 0.463, *t* = 4.519, *p* < 0.001), shallow cognitive engagement (*β* = 0.397, *t* = 3.864, *p* < 0.001), flow (*β* = 0.363, *t* = 5.295, *p* < 0.001), well-being (*β* = 0.308, *t* = 2.864, *p* < 0.001). Therefore, the H1 was supported. However, the predicting role of academic self-concept on both deep and shallow cognitive engagement was not observed, and the effect of flow on well-being was also unsupported.

**Table 7:** Assessment of the structural model.

Paths	Significance test of hypothesis				95% CI		Conclusion	*f* ^2^
	Estimate	STDEV	*t*	*p*	Lower	Upper		
ASC → DCE	0.128	0.106	1.202	0.229	−0.074	0.341	Not support	0.011
ASC → SCE	0.132	0.105	1.258	0.208	−0.072	0.341	Not support	0.010
ASC → Flow	0.550	0.064	8.544	***	0.422	0.675	Support	0.273
ASC → WB	0.465	0.116	4.007	***	0.240	0.686	Support	0.124
EOLE → ASC	0.902	0.015	60.45	***	0.870	0.928	Support	4.363
EOLE → DCE	0.463	0.102	4.519	***	0.257	0.655	Support	0.167
EOLE → SCE	0.397	0.103	3.864	***	0.194	0.592	Support	0.106
EOLE → Flow	0.363	0.069	5.295	***	0.230	0.501	Support	0.119
EOLE → WB	0.308	0.107	2.864	**	0.098	0.516	Support	0.062
Flow → DCE	0.332	0.101	3.277	**	0.150	0.548	Support	0.106
Flow → SCE	0.375	0.108	3.481	**	0.180	0.598	Support	0.117
Flow → WB	0.120	0.091	1.328	0.184	−0.051	0.307	Not support	0.012

**p < 0.01; ***p < 0.001. STDEV, standard deviation; 95% CI, 95% confidence intervals.

**Table 8:** *R*^2^ and *Q*^2^ assessment of the structural model.

Constructs	*R* ^2^	*Q*^2^ (=1-SSE/SSO)
ASC	0.814	0.778
DCE	0.786	0.693
SCE	0.752	0.668
Flow	0.794	0.671
WB	0.745	0.675

### 4.4 Mediating effect analysis

From Table 7, most of the direct effects among EOLE, academic self-concept, flow, deep and shallow cognitive engagement, and well-being were significant. Therefore, mediating effect analysis of academic self-concept and flow between EOLE and, cognitive engagement and well-being was further conducted. From Table 9, academic self-concept significantly mediated the impact of EOLE on well-being (effect size = 0.419, *p* < 0.001, [0.222, 0.623]). However, the indirect effects of EOLE on both deep (effect size = 0.115, *p* > 0.05, [−0.064, 0.301]) and shallow cognitive engagement (effect size = 0.119, *p* > 0.05, [−0.067, 0.301]) were not significant. Therefore, the hypothesis H2c was supported, while H2a and H2b were not. Interestingly, flow significantly mediated the relationship between EOLE and both deep (effect size = 0.120, *p* < 0.01, [0.053, 0.215]) and shallow cognitive engagement (effect size = 0.136, *p* < 0.01, [0.061, 0.242]). However, the indirect effects of EOLE on well-being (effect size = 0.044, *p* > 0.05, [−0.016, 0.120]) were not observed. Therefore, the hypotheses H3a and H3b were supported, while H3c was not. Furthermore, the sequential mediating effects of academic self-concept and flow sequentially in the process from EOLE to both deep (effect size = 0.165, *p* < 0.01, [0.066, 0.299]) and shallow cognitive engagement (effect size = 0.186, *p* < 0.01, [0.080, 0.322]) were statistically significant. However, the effect of EOLE on well-being was not sequentially mediated by academic self-concept and flow (effect size = 0.060, *p* > 0.05, [−0.020, 0.163]). Therefore, the hypotheses H4a and H4b were supported, while H4c was not.

**Table 9:** Mediating effect analysis.

Paths	Significance test of hypothesis				95% CI	
	Original Sample	STDEV	*t*	*p*	Lower	Upper
**Total Effect**						
EOLE → DCE	0.863	0.022	39.021	0.000	0.816	0.904
EOLE → SCE	0.838	0.025	33.614	0.000	0.786	0.883
EOLE → WB	0.830	0.026	31.865	0.000	0.776	0.878
Specific indirect effect						
EOLE → ASC → DCE	0.115	0.094	1.227	0.220	−0.064	0.301
EOLE → ASC → SCE	0.119	0.093	1.277	0.202	−0.067	0.301
EOLE → ASC → WB	0.419	0.102	4.116	***	0.222	0.623
EOLE → Flow → DCE	0.120	0.041	2.910	**	0.053	0.215
EOLE → Flow → SCE	0.136	0.047	2.907	**	0.061	0.242
EOLE → Flow → WB	0.044	0.034	1.285	0.199	−0.016	0.120
EOLE → ASC → Flow → DCE	0.165	0.059	2.768	**	0.066	0.299
EOLE → ASC → Flow → SCE	0.186	0.062	3.000	**	0.080	0.322
EOLE → ASC → Flow → WB	0.060	0.046	1.288	0.198	−0.020	0.163
Direct effect						
EOLE → DCE	0.463	0.102	4.519	0.000	0.257	0.655
EOLE → SCE	0.397	0.103	3.864	0.000	0.194	0.592
EOLE → WB	0.308	0.107	2.864	0.004	0.098	0.516

**p < 0.01; ***p < 0.001. STDEV, standard deviation; 95% CI, 95% confidence interval.

## 5 Discussion

The purpose of this study was to explore the impact of EOLE on cognitive engagement and well-being through academic self-concept and flow. Results showed that EOLE had direct influences on academic self-concept, flow, deep and shallow cognitive engagement, and well-being. Academic self-concept had a significant effect on well-being, while its effects on both deep and shallow cognitive engagement were not observed. Conversely, flow experience could significantly predict deep and shallow cognitive engagement, while its effect on well-being was not supported. In addition, the study also found that academic self-concept mediated the significant relationship between EOLE and well-being. Effects of EOLE on both deep and shallow cognitive engagement were mediated. Furthermore, academic self-concept and flow were sequential mediators of EOLE on both deep and shallow cognitive engagement.

Specifically, EOLE could significantly affected academic self-concept, flow, deep and shallow cognitive engagement, and well-being, which is consistent with previous research findings (e.g., Esteban-Millat et al., 2014; Kumi-Yeboah et al., 2018; Liu and Duan, 2022; Stanton et al., 2016). This meant that the perceived quality of online learning by students significantly influenced their overall learning experience. When the learning environment integrated competence beliefs, motivation, and meaning into students’ engagement to meet their academic expectations, students exhibited a greater sense of academic control and value (Chen and Lu, 2022), enabling them to fully immerse themselves in tasks and experience the enjoyment of learning itself (Yang et al., 2025). In line with SCT, individuals are neither fully autonomous agents nor mere passive transmitters of environmental influences; instead, they actively contribute to their own motivation and behavior within a triadic reciprocal causation system (Bandura, 1989). As expected, this study confirmed H1 that EOLE significantly influenced deep and shallow cognitive engagement, and well-being. Previous literature has also explored the relationship among EOLE, deep and shallow engagement, and well-being. For instance, Guo et al. (2023) explored the relationship between cognitive engagement and interaction level in online learning environments. The findings indicated that the quality of interactions in online learning environments was strongly associated with cognitive engagement. Similiarly, Caprara and Caprara (2022) argued that an optimal virtual learning environment had offered students both academic and emotional support while promoting their engagement and motivation. These findings indicate that positive elements in online learning environments, including interaction quality, technological and social support, were critical for designing effective and engaging online learning experiences (Cockerham et al., 2021; Huang and Zhang, 2022).

Students’ academic self-concept had significant effects on their flow experience. This result confirmed the view of Lesmana (2019) that academic self-concept and academic self-efficacy contribute to college students’ flow state and GPA. The result was also in line with Jackson et al. (2001) that self-concept and use of psychological skills were positively correlated with the flow state of athletes. Specifically, tasks with appropriate challenges and opportunities for skill development drove individuals to immerse themselves in them, experiencing engagement and satisfaction (Meng and Wang, 2025). Therefore, students should be confident enough in their abilities, specifically when facing complex and challenging tasks, which could contribute to their psychological experience in the learning process (De Manzano et al., 2010). Moreover, this study demonstrated H2c that academic self-concept mediated the relationship between EOLE and well-being. This finding was consistent with Lazarides and Raufelder’s (2021) research results, which showed that mathematical self-concept is positively correlated with students’ enjoyment in mathematics, but negatively correlated with anxiety. This suggested that when students perceived themselves as competent and proficient in their academic endeavors, they tended to experience greater life satisfaction, happiness, and sense of purpose (Zhang, 2024). Another, according to self-determination theory, individual growth and development depend on the satisfaction of basic psychological needs (Ryan and Deci, 2000). Individuals may feel a sense of accomplishment if competency needs could be satisfied (Niemiec and Ryan, 2009), which contributes to individual psychological well-being. That is, when designing strategies for improving students’ well-being, personal internal factors such as self-concept and positivity should be taken into account (McCullough et al., 2000).

However, contrary to our expectations, the H2a and H2b, the mediating role of academic self-concept in the relationships between EOLE and, deep and shallow cognitive engagement, were not significant. This finding is inconsistent with previous research showing that EOLE positively influence academic self-concept, which in turn affects deep and surface learning (Chen et al., 2015; Zhang et al., 2022). A possible reason may be that the online course overly emphasized the rehearsal elements of cognitive learning strategies, where rehearsals simply repeat information and may not be closely related to students’ actual competence (Lohbeck and Moschner, 2022). In addition, students with a high academic self-concept were inclined to believe they can effectively complete tasks they perceive as very easy, resulting in reduced effort and a lack of deep learning during task accomplishment (Schunk, 2012). Therefore, future online courses should prioritize fostering students’ interest in tasks and establishing attainable yet challenging goals to enhance students’ use of elaboration strategies (Lohbeck and Moschner, 2022).

Students’ flow experience mediated the relationship between EOLE and deep, shallow cognitive engagement. This result was to some extent consistent with the findings of Thomas and Baral (2023) that compared with traditional and test-based instructional designs, gamified instructional designs could significantly influence students’ learning engagement through a high level of flow experience. The viewpoints of Engeser et al. (2021) also provided support for this result that the state of flow, in the absence of extrinsic rewards, enabled individuals to be highly engaged in activities. This indicated that students who enjoyed the environment were more actively engaged in the learning environment and strived toward their self-determined goals (Özhan and Kocadere, 2020). However, the H3c that the mediating effect of flow in the relationship between EOLE and well-being was not observed. This conflicted with the findings of Lynch and Troy (2021) that flow significantly predicted an increase in positive emotions and a decrease in negative emotions in students after the various art classes across all 4 weeks. The possible reason may be that monotonous work could also produce a flow experience (Ilies et al., 2017), but it did not necessarily make it enjoyable and maintain well-being. In other words, Students’ engagement in this online course is driven by obsessive passion, as certain contingencies, such as the course being linked to their academic performance, they have to participate due to limited control over such contingencies, potentially resulting in diminished well-being (Philippe et al., 2009). Therefore, learning activities should be simultaneously challenging, enjoyable, and achievable (Kaya and Ercag, 2023), which could afford students’ psychological satisfaction and well-being in the process of completing tasks.

## 6 Implications

### 6.1 Theoretical implications

This study had several theoretical implications for improving cognitive engagement and well-being in online learning environments. To the best of our knowledge, research on the roles of academic self-concept and flow from EOLE to cognitive engagement and well-being remains unclear. This study highlighted the importance of students’ cognitive engagement and well-being in EOLE, and confirmed the sequential mediating role of academic self-concept and flow between EOLE and cognitive engagement. In addition, the results of this study verified the view of SCT (Bandura, 1991) that personal factors could contribute to students’ behavior in a flexible environment to achieve positive academic outcomes. Therefore, this study enriched the theoretical posits in a specific online learning environment.

### 6.2 Practical implications

This study also provided several practical implications for teachers to design and implement an engaged and friendly online learning environment. Firstly, EOLE significantly influences students’ academic self-concept, flow, deep and shallow cognitive engagement, and well-being. Online learning environments can facilitate synchronous or asynchronous interactions and communication between teachers and students with the learning materials and other members of the learning community (Ferrer et al., 2022). Therefore, teachers can take advantage of the convenience of online learning environments to assist students. For instance, in the online classroom, teachers should actively encourage students to participate in group discussions and interactions with peers to improve students’ motivation, offer timely feedback on students’ homework after class, and provide students with rich online learning resources to help them improve their learning efficiency. Secondly, the present study confirmed the sequential mediating role of academic self-concept and flow between EOLE and, deep and shallow cognitive engagement. Therefore, when designing tasks, teachers should consider both the actual competence of the students and the difficulty of the task (De Manzano et al., 2010), so that the task is challenging as well as the students can complete the task with effort. Finally, this study was not only concerned with students’ learning but also their psychological well-being. Teachers should take a positive approach to education and give students more care and support so that they can experience emotional warmth (Zhao et al., 2024). If students have psychological problems, teachers should communicate with their parents on time and work together to help students overcome their difficulties.

## 7 Conclusion, limitations, and future research

The present study examined the effect of EOLE on cognitive engagement and well-being. The findings demonstrated a sequential mediating role of academic self-concept and flow in predicting the power of EOLE on cognitive engagement.

While these findings provided some valuable implications, some limitations remained. Firstly, the cross-sectional research design in this study cannot test causality. Future research can combine cross-sectional and longitudinal research to explore causal relationships between variables. Secondly, this study only considered cognitive engagement in the online learning environment, and empirical studies showed that other dimensions of student engagement also affect students’ academic outcomes (Fan and Tian, 2022; Gunness et al., 2023). Therefore, future research should consider students’ emotional, behavioral, and agentic engagement so that students could optimally engage in online learning activities. Finally, the sample size of participants was insufficient to generalize this finding to other learning contexts. Future research should select more students from different school contexts to enhance the generalizability of the conclusion.

## Data Availability

The raw data supporting the conclusions of this article will be made available by the authors, without undue reservation.

## Footnotes

^1^ https://www.wjx.cn/

## References

[ref1] Alarifi B. N. Song S. (2024). Online vs in-person learning in higher education: effects on student achievement and recommendations for leadership. Humanit. Soc. Sci. Commun. 11, 1–8. doi: 10.1057/s41599-023-02590-1

[ref2] Alon L. Sung S. Cho J. Kizilcec R. F. (2023). From emergency to sustainable online learning: changes and disparities in undergraduate course grades and experiences in the context of COVID-19. Comput. Educ. 203:104870. doi: 10.1016/j.compedu.2023.104870

[ref3] Arens A. K. Jansen M. Preckel F. Schmidt I. Brunner M. (2021). The structure of academic self-concept: a methodological review and empirical illustration of central models. Rev. Educ. Res. 91, 34–72. doi: 10.3102/0034654320972186

[ref4] Bandura A. (1989). Human agency in social cognitive theory. Am. Psychol. 44, 1175–1184. doi: 10.1037/0003-066X.44.9.1175, PMID: 2782727

[ref5] Bandura A. (1991). Social cognitive theory of self-regulation. Organ. Behav. Hum. Decis. Process. 50, 248–287. doi: 10.1016/0749-5978(91)90022-L

[ref6] Barrot J. S. Llenares I. I. Del Rosario L. S. (2021). Students’ online learning challenges during the pandemic and how they cope with them: the case of the Philippines. Educ. Inf. Technol. 26, 7321–7338. doi: 10.1007/s10639-021-10589-x, PMID: 34075300 PMC8162157

[ref7] Barthelmäs M. Keller J. (2021). Antecedents, boundary conditions and consequences of flow. Advances in flow research. Cham: Springer International Publishing, 71–107.

[ref8] Bonaiuto M. Mao Y. Roberts S. Psalti A. Ariccio S. Ganucci Cancellieri U.. (2016). Optimal experience and personal growth: flow and the consolidation of place identity. Front. Psychol. 7:1654. doi: 10.3389/fpsyg.2016.01654, PMID: 27872600 PMC5097910

[ref9] Bringula R. Reguyal J. J. Tan D. D. Ulfa S. (2021). Mathematics self-concept and challenges of learners in an online learning environment during COVID-19 pandemic. Smart. Learn. Environ. 8:22. doi: 10.1186/s40561-021-00168-5, PMID: 40477819 PMC8520328

[ref10] Brunner M. Keller U. Dierendonck C. Reichert M. Ugen S. Fischbach A.. (2010). The structure of academic self-concepts revisited: the nested Marsh/Shavelson model. J. Educ. Psychol. 102, 964–981. doi: 10.1037/a0019644

[ref11] Buhs E. S. (2005). Peer rejection, negative peer treatment, and school adjustment: self-concept and classroom engagement as mediating processes. J. Sch. Psychol. 43, 407–424. doi: 10.1016/j.jsp.2005.09.001

[ref12] Buijs M. Admiraal W. (2013). Homework assignments to enhance student engagement in secondary education. Eur. J. Psychol. Educ. 28, 767–779. doi: 10.1007/s10212-012-0139-0

[ref13] Caprara L. Caprara C. (2022). Effects of virtual learning environments: a scoping review of literature. Educ. Inf. Technol. 27, 3683–3722. doi: 10.1007/s10639-021-10768-w, PMID: 34629934 PMC8492824

[ref14] Carillo K. D. (2010). “Social cognitive theory in is research–literature review, criticism, and research agenda” in Information systems, technology and management: 4th international conference, ICISTM 2010, Bangkok, Thailand, March 11–13, 2010. Proceedings 4. Eds. S. K. Prasad, H. M. Vin, S. Sahni, M. P. Jaiswal and B. Thipakorn. (Springer Berlin Heidelberg), 20–31.

[ref15] Céspedes C. Rubio A. Viñas F. Cerrato S. M. Lara-Órdenes E. Ríos J. (2021). Relationship between self-concept, self-efficacy, and subjective well-being of native and migrant adolescents. Front. Psychol. 11:620782. doi: 10.3389/fpsyg.2020.620782, PMID: 33584455 PMC7873051

[ref16] Chen B. H. Chiu W. C. Wang C. C. (2015). The relationship among academic self-concept, learning strategies, and academic achievement: a case study of national vocational college students in Taiwan via SEM. Asia-Pac. Educ. Res. 24, 419–431. doi: 10.1007/s40299-014-0194-1

[ref17] Chen M. Chai C. S. Jong M. S. Y. Chao G. C. N. (2021). Modeling learners’ self-concept in Chinese descriptive writing based on the affordances of a virtual reality-supported environment. Educ. Inf. Technol. 26, 6013–6032. doi: 10.1007/s10639-021-10582-4

[ref18] Chen X. Lu L. (2022). How classroom management and instructional clarity relate to students' academic emotions in Hong Kong and England: a multi-group analysis based on the control-value theory. Learn. Individ. Differ. 98:102183. doi: 10.1016/j.lindif.2022.102183

[ref19] Chin W. W. (1998). “The partial least squares approach to structural equation modeling” in Modern methods for business research. Ed. G. A. Marcoulides. (New York: Lawrence Erlbaum Associates).

[ref20] Chiu T. K. (2022). Applying the self-determination theory (SDT) to explain student engagement in online learning during the COVID-19 pandemic. J. Res. Technol. Educ. 54, S14–S30. doi: 10.1080/15391523.2021.1891998

[ref21] Chiu T. K. Lin T. J. Lonka K. (2021). Motivating online learning: the challenges of COVID-19 and beyond. Asia-Pac. Educ. Res. 30, 187–190. doi: 10.1007/s40299-021-00566-w

[ref22] Cockerham D. Lin L. Ndolo S. Schwartz M. (2021). Voices of the students: adolescent well-being and social interactions during the emergent shift to online learning environments. Educ. Inf. Technol. 26, 7523–7541. doi: 10.1007/s10639-021-10601-4, PMID: 34149300 PMC8202218

[ref23] Cole A. W. Lennon L. Weber N. L. (2021). Student perceptions of online active learning practices and online learning climate predict online course engagement. Interact. Learn. Environ. 29, 866–880. doi: 10.1080/10494820.2019.1619593

[ref24] Coutts J. J. Al-Kire R. L. Weidler D. J. (2023). I can see (myself) clearly now: exploring the mediating role of self-concept clarity in the association between self-compassion and indicators of well-being. PLoS One 18:e0286992. doi: 10.1371/journal.pone.0286992, PMID: 37390089 PMC10313035

[ref25] Craik F. I. Lockhart R. S. (1972). Levels of processing: a framework for memory research. J. Verbal Learn. Verbal Behav. 11, 671–684. doi: 10.1016/S0022-5371(72)80001-X

[ref27] Csikszentmihalyi M. Csikzentmihaly M. (1990). Flow: the psychology of optimal experience, vol. 1990. New York: Harper & Row, 1.

[ref28] Czikszentmihalyi M. (1990). Flow: the psychology of optimal experience. New York: Harper & Row, 75–77.

[ref26] Csikszentmihalhi M. (2020). Finding flow: The psychology of engagement with everyday life. Hachette UK.

[ref29] De Manzano Ö. Theorell T. Harmat L. Ullén F. (2010). The psychophysiology of flow during piano playing. Emotion 10, 301–311. doi: 10.1037/a0018432, PMID: 20515220

[ref30] Deci E. L. Ryan R. M. (2008). Hedonia, eudaimonia, and well-being: an introduction. J. Happiness Stud. 9, 1–11. doi: 10.1007/s10902-006-9018-1

[ref31] Diener E. (1984). Subjective well-being. Psychol. Bull. 95, 542–575. doi: 10.1037/0033-2909.95.3.542, PMID: 6399758

[ref32] Diener E. Wirtz D. Tov W. Kim-Prieto C. Choi D. W. Oishi S.. (2010). New well-being measures: short scales to assess flourishing and positive and negative feelings. Soc. Indic. Res. 97, 143–156. doi: 10.1007/s11205-009-9493-y

[ref33] Engeser S. Schiepe-Tiska A. Peifer C. (2021). Historical lines and an overview of current research on flow. Advances in flow research, Springer Cham, 1–29.

[ref34] Esteban-Millat I. Martínez-López F. J. Huertas-García R. Meseguer A. Rodríguez-Ardura I. (2014). Modelling students' flow experiences in an online learning environment. Comput. Educ. 71, 111–123. doi: 10.1016/j.compedu.2013.09.012

[ref35] Fan J. Tian M. (2022). Influence of online learning environment and student engagement on international students’ sustainable Chinese learning. Sustainability 14:11106. doi: 10.3390/su141711106

[ref36] Ferrer J. Ringer A. Saville K. Parris A. Kashi K. (2022). Students’ motivation and engagement in higher education: the importance of attitude to online learning. High. Educ. 83, 317–338. doi: 10.1007/s10734-020-00657-5

[ref37] Fiddiyasari A. Pustika R. (2021). Students’ motivation in English online learning during Covid-19 pandemic at SMA Muhammadiyah Gadingrejo. J. Engl. Lang. Teach. Learn. 2, 57–61. doi: 10.33365/jeltl.v2i2.1217

[ref38] Fredricks J. A. Blumenfeld P. C. Paris A. H. (2004). School engagement: potential of the concept, state of the evidence. Rev. Educ. Res. 74, 59–109. doi: 10.3102/00346543074001059

[ref40] Goh T. T. Yang B. (2021). The role of e-engagement and flow on the continuance with a learning management system in a blended learning environment. Int. J. Educ. Technol. High. Educ. 18:49. doi: 10.1186/s41239-021-00285-8

[ref41] Gray J. A. DiLoreto M. (2016). The effects of student engagement, student satisfaction, and perceived learning in online learning environments. Int. J. Educ. Leadersh. Prep. 11:n1. Available online at: https://eric.ed.gov/?id=EJ1103654

[ref42] Greene B. A. Miller R. B. (1996). Influences on achievement: goals, perceived ability, and cognitive engagement. Contemp. Educ. Psychol. 21, 181–192. doi: 10.1006/ceps.1996.0015 8979871

[ref43] Gunness A. Matanda M. J. Rajaguru R. (2023). Effect of student responsiveness to instructional innovation on student engagement in semi-synchronous online learning environments: the mediating role of personal technological innovativeness and perceived usefulness. Comput. Educ. 205:104884. doi: 10.1016/j.compedu.2023.104884

[ref44] Guo J. P. Yang L. Y. Zhang J. Gan Y. J. (2022). Academic self-concept, perceptions of the learning environment, engagement, and learning outcomes of university students: relationships and causal ordering. High. Educ. 83, 809–828. doi: 10.1007/s10734-021-00705-8

[ref45] Guo L. Du J. Zheng Q. (2023). Understanding the evolution of cognitive engagement with interaction levels in online learning environments: insights from learning analytics and epistemic network analysis. J. Comput. Assist. Learn. 39, 984–1001. doi: 10.1111/jcal.12781

[ref47] Hair J. F. Sarstedt M. Ringle C. M. Mena J. A. (2012). An assessment of the use of partial least squares structural equation modeling in marketing research. J. Acad. Mark. Sci. 40, 414–433. doi: 10.1007/s11747-011-0261-6

[ref48] Heilporn G. Lakhal S. Bélisle M. (2022). Examining effects of instructional strategies on student engagement in blended online courses. J. Comput. Assist. Learn. 38, 1657–1673. doi: 10.1111/jcal.12701

[ref49] Heo H. Bonk C. J. Doo M. Y. (2021). Enhancing learning engagement during COVID-19 pandemic: self-efficacy in time management, technology use, and online learning environments. J. Comput. Assist. Learn. 37, 1640–1652. doi: 10.1111/jcal.12603

[ref50] Hoi V. N. (2022). Measuring students’ perception of an engaging online learning environment: an argument-based scale validation study. Educ. Technol. Res. Dev. 70, 2033–2062. doi: 10.1007/s11423-022-10155-3

[ref51] Huang L. Zhang T. (2022). Perceived social support, psychological capital, and subjective well-being among college students in the context of online learning during the COVID-19 pandemic. Asia-Pac. Educ. Res. 31, 563–574. doi: 10.1007/s40299-021-00608-3

[ref52] Huppert F. A. (2009). Psychological well-being: evidence regarding its causes and consequences. Appl. Psychol. Health Well-Being 1, 137–164. doi: 10.1111/j.1758-0854.2009.01008.x

[ref53] Ilies R. Wagner D. Wilson K. Ceja L. Johnson M. DeRue S.. (2017). Flow at work and basic psychological needs: effects on well-being. Appl. Psychol. 66, 3–24. doi: 10.1111/apps.12075

[ref54] Jackson S. A. Thomas P. R. Marsh H. W. Smethurst C. J. (2001). Relationships between flow, self-concept, psychological skills, and performance. J. Appl. Sport Psychol. 13, 129–153. doi: 10.1080/104132001753149865

[ref55] Jiang L. Zhou N. Yang Y. (2024). Student motivation and engagement in online language learning using virtual classrooms: interrelationships with support, attitude and learner readiness. Educ. Inf. Technol. 29, 17119–17143. doi: 10.1007/s10639-024-12514-4, PMID: 41014363

[ref56] Kaya O. S. Ercag E. (2023). The impact of applying challenge-based gamification program on students’ learning outcomes: academic achievement, motivation and flow. Educ. Inf. Technol. 28, 10053–10078. doi: 10.1007/s10639-023-11585-z, PMID: 36691635 PMC9850335

[ref57] Keyes C. L. M. (1998). Social well-being. Soc. Psychol. Q. 61, 121–140. doi: 10.2307/2787065

[ref58] Kumi-Yeboah A. Dogbey J. Yuan G. (2018). Exploring factors that promote online learning experiences and academic self-concept of minority high school students. J. Res. Technol. Educ. 50, 1–17. doi: 10.1080/15391523.2017.1365669, PMID: 40989069

[ref59] Landhäußer A. Keller J. (2012). Flow and its affective, cognitive, and performance-related consequences. Advances in flow research, Springer Cham, 65–85.

[ref60] Lazarides R. Raufelder D. (2021). Control-value theory in the context of teaching: does teaching quality moderate relations between academic self-concept and achievement emotions? Br. J. Educ. Psychol. 91, 127–147. doi: 10.1111/bjep.12352, PMID: 32369196

[ref61] Lesmana T. (2019). Hubungan antara academic self-concept dan academic self-efficacy dengan flow pada mahasiswa Universitas X. J. Psikol. Ulayat. 6, 117–134. doi: 10.24854/jpu90

[ref62] Lin L. Wang J. Meng X. (2022). Influencing factors of learners’ cognitive engagement in an online learning environment: a PST model. Int. J. Emerg. Technol. Learn. 17:127. doi: 10.3991/ijet.v17i17.33851

[ref63] Lin Y. L. Wang W. T. Hsieh M. J. (2024). The effects of students’ self-efficacy, self-regulated learning strategy, perceived and actual learning effectiveness: a digital game-based learning system. Educ. Inf. Technol. 29, 22213–22245. doi: 10.1007/s10639-024-12700-4, PMID: 41014363

[ref64] Liu L. Duan Z. (2022). Influences of environmental perception on individual cognitive engagement in online learning: the mediating effect of self-efficacy. Int. J. Emerg. Technol. Learn. 17, 66–78. doi: 10.3991/ijet.v17i04.29221

[ref65] Liu Z. Zhang N. Peng X. Liu S. Yang Z. (2023). Students’ social-cognitive engagement in online discussions. Educ. Technol. Soc. 26, 1–15. doi: 10.30191/ETS.202301_26(1).0001

[ref66] Lockee B. B. (2021). Online education in the post-COVID era. Nat. Electron. 4, 5–6. doi: 10.1038/s41928-020-00534-0

[ref67] Lohbeck A. Moschner B. (2022). Motivational regulation strategies, academic self-concept, and cognitive learning strategies of university students: does academic self-concept play an interactive role? Eur. J. Psychol. Educ. 37, 1217–1236. doi: 10.1007/s10212-021-00583-9

[ref68] Lu G. Xie K. Liu Q. (2023). An experience-sampling study of between-and within-individual predictors of emotional engagement in blended learning. Learn. Individ. Differ. 107:102348. doi: 10.1016/j.lindif.2023.102348

[ref69] Lynch J. M. Troy A. S. (2021). The role of nonduality in the relationship between flow states and well-being. Mindfulness 12, 1639–1652. doi: 10.1007/s12671-021-01627-3

[ref70] Mao Y. Luo X. Wang S. Mao Z. Xie M. Bonaiuto M. (2024). Flow experience fosters university students' well-being through psychological resilience: a longitudinal design with cross-lagged analysis. Br. J. Educ. Psychol. 94, 518–538. doi: 10.1111/bjep.12661, PMID: 38238106

[ref71] Marsh H. W. Shavelson R. (1985). Self-concept: its multifaceted, hierarchical structure. Educ. Psychol. 20, 107–123. doi: 10.1207/s15326985ep2003_1

[ref72] Matovu M. (2014). A structural equation modelling of the academic self-concept scale. IEJEE 6, 185–198. Available online at: https://eric.ed.gov/?id=EJ1053584

[ref73] McCullough G. Huebner E. S. Laughlin J. E. (2000). Life events, self-concept, and adolescents' positive subjective well-being. Psychol. Sch. 37, 281–290. doi: 10.1002/(SICI)1520-6807(200005)37:3<281::AID-PITS8>3.0.CO;2-2

[ref74] Meng L. Wang X. (2025). The compensation effect after competence frustration: utilizing the lifespan developmental perspective on flow theory. J. Happiness Stud. 26, 1–28. doi: 10.1007/s10902-025-00936-x39664799

[ref75] Mesurado B. Cristina Richaud M. José Mateo N. (2016). Engagement, flow, self-efficacy, and eustress of university students: a cross-national comparison between the Philippines and Argentina. J. Psychol. 150, 281–299. doi: 10.1080/00223980.2015.1024595, PMID: 25915707

[ref76] Miller R. B. Greene B. A. Montalvo G. P. Ravindran B. Nichols J. D. (1996). Engagement in academic work: the role of learning goals, future consequences, pleasing others, and perceived ability. Contemp. Educ. Psychol. 21, 388–422. doi: 10.1006/ceps.1996.0028, PMID: 8979871

[ref77] Murphy E. R. (2023). Hope and well-being. Curr. Opin. Psychol. 50:101558. doi: 10.1016/j.copsyc.2023.101558, PMID: 36822123

[ref78] Niemiec C. P. Ryan R. M. (2009). Autonomy, competence, and relatedness in the classroom: applying self-determination theory to educational practice. Theory Res. Educ. 7, 133–144. doi: 10.1177/1477878509104318

[ref80] Özhan Ş. Ç. Kocadere S. A. (2020). The effects of flow, emotional engagement, and motivation on success in a gamified online learning environment. J. Educ. Comput. Res. 57, 2006–2031. doi: 10.1177/0735633118823159

[ref81] Pan X. (2022). Exploring the multidimensional relationships between educational situation perception, teacher support, online learning engagement, and academic self-efficacy in technology-based language learning. Front. Psychol. 13:1000069. doi: 10.3389/fpsyg.2022.1000069, PMID: 36467143 PMC9714665

[ref82] Park J. Parsons D. Ryu H. (2010). To flow and not to freeze: applying flow experience to mobile learning. IEEE Trans. Learn. Technol. 3, 56–67. doi: 10.1109/TLT.2010.1

[ref83] Philippe F. L. Vallerand R. J. Lavigne G. L. (2009). Passion does make a difference in people's lives: a look at well-being in passionate and non-passionate individuals. Appl. Psychol. Health Well-Being 1, 3–22. doi: 10.1111/j.1758-0854.2008.01003.x

[ref84] Pritikin J. N. Schmidt K. M. (2022). Physical activity flow propensity: scale development using exploratory factor analysis with paired comparison indicators. Int. J. Appl. Posit. Psychol. 7, 327–354. doi: 10.1007/s41042-022-00071-5

[ref85] Rheinberg F. Manig Y. Kliegl R. Engeser S. Vollmeyer R. (2007). Flow bei der Arbeit, doch Glück in der Freizeit: Zielausrichtung, Flow und Glücksgefühle. Zeitschrift für Arbeits- und Organisationspsychologie A&O 51, 105–115. doi: 10.1026/0932-4089.51.3.105

[ref86] Rohan R. Dutsinma F. L. I. Pal D. Funilkul S. (2022). “Applying the stimulus organism response framework to explain student’s academic self-concept in online learning during the COVID-19 pandemic” in Advances in data and information sciences: Proceedings of ICDIS 2022 (Singapore: Springer Nature Singapore), 373–384.

[ref87] Ryan R. M. Deci E. L. (2000). Self-determination theory and the facilitation of intrinsic motivation, social development, and well-being. Am. Psychol. 55:68. doi: 10.1037/0003-066X.55.1.68 11392867

[ref88] Salas-Pilco S. Z. Yang Y. Zhang Z. (2022). Student engagement in online learning in Latin American higher education during the COVID-19 pandemic: a systematic review. Br. J. Educ. Technol. 53, 593–619. doi: 10.1111/BJET.13190, PMID: 35600418 PMC9111674

[ref89] Sarstedt M. Hair J. J. F. Cheah J. H. Becker J. M. Ringle C. M. (2019). How to specify, estimate, and validate higher-order constructs in PLS-SEM. Australas. Mark. J. 27, 197–211. doi: 10.1016/j.ausmj.2019.05.003

[ref90] Sarstedt M. Ringle C. M. Hair J. F. (2021). “Partial least squares structural equation modeling” in Handbook of market research. Eds. C. Homburg, M. Klarmann and A. E. Vomberg. (Cham: Springer International Publishing), 587–632.

[ref91] Schnitzler K. Holzberger D. Seidel T. (2021). All better than being disengaged: student engagement patterns and their relations to academic self-concept and achievement. Eur. J. Psychol. Educ. 36, 627–652. doi: 10.1007/s10212-020-00500-6

[ref92] Schunk D. H. (2012). Social cognitive theory United States of America, American Psychological Association.

[ref93] Schunk D. H. DiBenedetto M. K. (2016). “Self-efficacy theory in education” in Handbook of motivation at school. Eds. K. R. Wentzel and D. B. Miele. (New York: Routledge), 34–54.

[ref94] Schunk D. H. DiBenedetto M. K. (2020). Motivation and social cognitive theory. Contemp. Educ. Psychol. 60:101832. doi: 10.1016/j.cedpsych.2019.101832

[ref95] Schunk D. H. Usher E. L. (2012). “Social cognitive theory and motivation” in The Oxford handbook of human motivation, Ed. R. Ryan. (New York, Oxford University Press), vol. 2, 11–26.

[ref96] Shahzad M. F. Xu S. Zahid H. (2025). Exploring the impact of generative AI-based technologies on learning performance through self-efficacy, fairness & ethics, creativity, and trust in higher education. Educ. Inf. Technol. 30, 3691–3716. doi: 10.1007/s10639-024-12949-9

[ref97] Shi Y. Cheng Q. Wei Y. Tong M. Yao H. (2024). Understanding the effect of video conferencing learning environments on students' engagement: the role of basic psychological needs. J. Comput. Assist. Learn. 40, 288–305. doi: 10.1111/jcal.12880

[ref98] Shi Y. Tong M. Long T. (2021). Investigating relationships among blended synchronous learning environments, students’ motivation, and cognitive engagement: a mixed methods study. Comput. Educ. 168:104193. doi: 10.1016/j.compedu.2021.104193

[ref99] Shin N. (2006). Online learner’s ‘flow’experience: an empirical study. Br. J. Educ. Technol. 37, 705–720. doi: 10.1111/j.1467-8535.2006.00641.x

[ref100] Slack H. R. Priestley M. (2023). Online learning and assessment during the Covid-19 pandemic: exploring the impact on undergraduate student well-being. Assess. Eval. High. Educ. 48, 333–349. doi: 10.1080/02602938.2022.2076804

[ref101] Stanton A. Zandvliet D. Dhaliwal R. Black T. (2016). Understanding students' experiences of well-being in learning environments. High. Educ. Stud. 6, 90–99. doi: 10.5539/hes.v6n3p90

[ref102] Stark E. (2019). Examining the role of motivation and learning strategies in student success in online versus face-to-face courses. Online. Learn. 23, 234–251. doi: 10.24059/olj.v23i3.1556, PMID: 33692645

[ref103] Steinberg O. Kulakow S. Raufelder D. (2024). Academic self-concept, achievement, and goal orientations in different learning environments. Eur. J. Psychol. Educ. 39, 3893–3917. doi: 10.1007/s10212-024-00825-6

[ref104] Sugden N. Brunton R. MacDonald J. Yeo M. Hicks B. (2021). Evaluating student engagement and deep learning in interactive online psychology learning activities. Australas. J. Educ. Technol. 37, 45–65. doi: 10.14742/ajet.6632

[ref105] Suryaratri R. D. Komalasari G. Medellu G. I. (2022). The role of academic self-efficacy and social support in achieving academic flow in online learning. Int. J. Technol. Educ. Sci. 6, 164–177. doi: 10.46328/ijtes.345

[ref106] Tas Y. (2016). The contribution of perceived classroom learning environment and motivation to student engagement in science. Eur. J. Psychol. Educ. 31, 557–577. doi: 10.1007/s10212-016-0303-z

[ref107] Thomas N. J. Baral R. (2023). Mechanism of gamification: role of flow in the behavioral and emotional pathways of engagement in management education. Int. J. Manag. Educ. 21:100718. doi: 10.1016/j.ijme.2022.100718

[ref108] Tov W. (2018). Well-being concepts and components Salt Lake City, UT, Noba Scholar.

[ref109] Turk M. Heddy B. C. Danielson R. W. (2022). Teaching and social presences supporting basic needs satisfaction in online learning environments: how can presences and basic needs happily meet online? Comput. Educ. 180:104432. doi: 10.1016/j.compedu.2022.104432

[ref110] Wang Q. Wen Y. Quek C. L. (2023). Engaging learners in synchronous online learning. Educ. Inf. Technol. 28, 4429–4452. doi: 10.1007/s10639-022-11393-x, PMID: 36277511 PMC9574801

[ref111] Wang Y. Cao Y. Gong S. Wang Z. Li N. Ai L. (2022). Interaction and learning engagement in online learning: the mediating roles of online learning self-efficacy and academic emotions. Learn. Individ. Differ. 94:102128. doi: 10.1016/j.lindif.2022.102128

[ref112] Wasson L. T. Cusmano A. Meli L. Louh I. Falzon L. Hampsey M.. (2016). Association between learning environment interventions and medical student well-being: a systematic review. JAMA 316, 2237–2252. doi: 10.1001/jama.2016.17573, PMID: 27923091 PMC5240821

[ref113] Wu H. Guo Y. Yang Y. Zhao L. Guo C. (2021). A meta-analysis of the longitudinal relationship between academic self-concept and academic achievement. Educ. Psychol. Rev. 33, 1749–1778. doi: 10.1007/s10648-021-09600-1, PMID: 41014363

[ref114] Wu Y. Kang X. (2023). Perceived teacher support and EFL achievement: the mediating roles of academic enjoyment and self-concept. Int. J. Linguist. Transl. Stud. 4, 38–53. doi: 10.36892/ijlts.v4i2.320

[ref115] Yang Y. Chen J. Zhuang X. (2025). Self-determination theory and the influence of social support, self-regulated learning, and flow experience on student learning engagement in self-directed e-learning. Front. Psychol. 16:1545980. doi: 10.3389/fpsyg.2025.1545980, PMID: 40207130 PMC11979257

[ref116] Zhan Z. Mei H. (2013). Academic self-concept and social presence in face-to-face and online learning: perceptions and effects on students' learning achievement and satisfaction across environments. Comput. Educ. 69, 131–138. doi: 10.1016/j.compedu.2013.07.002

[ref117] Zhang D. Cao M. Tian Y. (2024). Avatar identification and internet gaming disorder among Chinese middle school students: the serial mediating roles of flow and self-concept clarity. Int. J. Ment. Health Addict. 22, 1194–1208. doi: 10.1007/s11469-022-00923-w

[ref118] Zhang H. (2024). Psychological wellbeing in Chinese university students: insights into the influences of academic self-concept, teacher support, and student engagement. Front. Psychol. 14:1336682. doi: 10.3389/fpsyg.2023.1336682, PMID: 38292520 PMC10824945

[ref119] Zhang S. Ma R. Wang Z. Li G. Fa T. (2022). Academic self-concept mediates the effect of online learning engagement on deep learning in online courses for Chinese nursing students: a cross-sectional study. Nurse Educ. Today 117:105481. doi: 10.1016/j.nedt.2022.105481, PMID: 35872403

[ref120] Zhao H. Wan L. Li Y. Zhang M. Zhao C. (2024). Parental psychological control and interpersonal Trust in Junior High School Students: serial mediating roles of shyness and interpersonal self-support. Psychol. Res. Behav. Manag. 17, 4087–4104. doi: 10.2147/PRBM.S478008, PMID: 39650087 PMC11622682

[ref121] Zhou J. Yu H. (2021). Contribution of social support to home-quarantined Chinese college students’ well-being during the COVID-19 pandemic: the mediating role of online learning self-efficacy and moderating role of anxiety. Soc. Psychol. Educ. 24, 1643–1662. doi: 10.1007/s11218-021-09665-4, PMID: 34720666 PMC8543427

[ref122] Zhu C. Van Winkel L. (2016). A virtual learning environment for the continuation of education and its relationship with the mental well-being of chronically ill adolescents. Educ. Psychol. 36, 1429–1442. doi: 10.1080/01443410.2014.992393

